# Risk Factors and Population-Attributable Fractions for Incident Hip Osteoarthritis

**DOI:** 10.1177/15563316231192461

**Published:** 2023-08-29

**Authors:** Jos Runhaar, Annemaria C. van Berkel, Rintje Agricola, Joyce van Meurs, Sita M. A. Bierma-Zeinstra

**Affiliations:** 1 Department of General Practice, Erasmus MC University Medical Center Rotterdam, Rotterdam, the Netherlands; 2 Department of Orthopedics & Sports Medicine, Erasmus MC University Medical Center Rotterdam, Rotterdam, the Netherlands; 3 Department of Internal Medicine, Erasmus MC University Medical Center Rotterdam, Rotterdam, the Netherlands; 4 Department of Orthopedic Surgery, St Anna Hospital, Geldrop, the Netherlands

**Keywords:** hip osteoarthritis, incidence, population-attributable fraction, prevention

## Abstract

**Background:**

Despite the huge burden of hip osteoarthritis (OA) and the lack of effective treatment, research into the primary prevention of hip OA is in its infancy.

**Purpose:**

We sought to evaluate risk factors for incident clinical and incident radiographic hip OA among middle-aged and older adults, to evaluate the importance of risk factors from a preventive perspective, and to estimate the percentage of new cases attributable to these risk factors.

**Methods:**

We retrospectively reviewed data from the Rotterdam study, an open-population cohort study of individuals aged 55 years or older. Data including baseline age, sex, body mass index, smoking status, education level, diagnosis of diabetes, C-reactive protein (CRP), cam morphology, acetabular dysplasia, radiographic thumb OA, radiographic hip OA, and hip pain were assessed for their association with incident clinical hip OA and incident radiographic hip OA separately, after 11 years of follow-up. The population-attributable fractions (PAFs) of statistically significant modifiable risk factors were calculated, as well.

**Results:**

New onset of clinical hip OA was seen in 19.9% (544 of 2729) and incident radiographic hip OA in 9.9% (329 of 3309). Female sex, education level below average (PAF 21.4%), and radiographic hip OA (PAF 3.4%) were statistically significantly associated with incident clinical hip OA. Female sex, age, overweight (PAF 20.0%), cam morphology (PAF 7.9%), acetabular dysplasia (PAF 3.6%), and radiographic thumb OA (PAF 4.7%) were statistically significantly associated with radiographic hip OA.

**Conclusions:**

Our retrospective analysis suggests that, from a primary prevention perspective, the most important modifiable risk factors among middle-aged and older individuals may be low educational level for incident clinical hip OA and overweight for incident radiographic hip OA. Further study is warranted.

## Introduction

Although research into the primary prevention of osteoarthritis (OA) in general, and of hip OA in particular, is in its infancy [7,22,27], the huge burden of hip osteoarthritis (OA) on patients, society, and the healthcare system, together with the rising prevalence and the small-to-moderate nonsurgical treatment effects [6,7,14], make efforts around the primary prevention of hip OA highly relevant [23,27].

It is essential to identify risk factors for OA development, to determine target populations for OA prevention efforts, and to develop potential interventions [22,23,27]. Nonmodifiable risk factors can be used to identify individuals at increased risk, while modifiable risk factors can be used to select patients and target treatments. A recent systematic review of multivariable prediction models for OA development in the general population underscored the lack of knowledge of risk factors for hip OA development [2]. Of the 31 identified risk-prediction models studied, only 4 had hip OA development as an outcome. Despite the clinical relevance of illness over disease (“OA disease” being pathologic changes in joint tissues and “OA illness” being symptoms of OA), all 4 identified hip OA prediction models used structural hip OA (eg, radiographic hip OA, hip arthroplasty, or both) as an outcome.

Studying risk factors for incident clinical hip OA is essential in furthering our knowledge around the potential treatment targets for OA prevention that could have a clinically relevant impact on disease burden. From a population perspective, looking beyond how strongly a risk factor is associated with OA development is also key. A helpful epidemiologic measure for this is the population-attributable fraction (PAF). PAF is defined as the fraction of all new cases of a particular disease in a population attributable to a specific exposure (risk factor) [17]. PAF is directly related to both the relative risk and the prevalence of a certain risk factor. For knee OA development, previous studies showed a PAF of 5% for previous knee injuries and a range of 8 to 50% for obesity [7]. Currently, no reports are available on PAFs for hip OA development.

Given these knowledge gaps, and the fact that there is a marked increase in the incidence of hip OA after 50 years of age [20], we had 2 aims: (1) to report on risk factors for hip OA development in an open population of middle-aged and older individuals, stratified for incident clinical hip OA and incident radiographic hip OA and (2) to calculate PAFs for statistically significant modifiable risk factors to explore their potential for future preventive interventions.

## Methods

For this study, we retrospectively reviewed data from the first 2 cohorts of the Rotterdam Study (RS) [9]. The RS is a prospective open-population cohort comprising people living in the Ommoord region, Rotterdam, the Netherlands. The first cohort (RS-I) started in 1989 with 7983 participants aged 55 years or older (78% of 10,215 invitees). The second cohort (RS-II) started in 2000 and had 3011 participants aged 55 years or older (67% of 4472 invitees). All participants gave written informed consent prior to participation, and the RS was approved by the Medical Ethics Committee of Erasmus MC.

At baseline, questionnaires were used to obtain age, sex, current hip joint symptoms (yes or no), smoking status (never, former, current), diabetes (yes or no), and education level (below average, above average). Height and weight were measured to calculate body mass index (BMI), which was categorized as normal/underweight (BMI < 25 kg/m^2^), overweight (BMI 25–30 kg/m^2^), and obese (BMI > 30 kg/m^2^). Venipuncture was performed to assess the serum C-reactive protein (CRP) [13]. Radiographs of the hand were obtained to establish the presence of thumb OA [15].

At baseline and each follow-up visit (every 3–6 years), anteroposterior pelvic radiographs of the hips were obtained and assessed using Kellgren and Lawrence (K&L) criteria [15]. Radiographic hip OA was defined as K&L grade 2 or greater. On baseline pelvic radiographs, acetabular dysplasia was determined using the center-edge angle of less than 20°, as defined by Wiberg [28]. Cam morphology was defined as an alpha angle greater than 60° [26].

Risk factors were selected based on the literature [24] and included sex, age, BMI categories, smoking status, education level, diabetes, CRP (≥10 vs < 10 mg/L), cam morphology, acetabular dysplasia, radiographic thumb OA, radiographic hip OA (only for incident clinical hip OA analyses), and hip pain (only for incident radiographic hip OA analyses). All risk factors were determined on a person level. The 2 primary outcome measures were self-reported incident hip pain (as a measure for clinical hip OA) in subjects with no hip pain at baseline and incident radiographic hip OA (K&L ≥ 2, including hip arthroplasty) in subjects with bilateral K&L less than grade 2 and no hip arthroplasty at baseline. Both outcome measures were determined on a person level, hence were defined as incidence in 1 or both hips. Baseline characteristics were determined using descriptive analyses and compared between selected subjects with and without available outcome measures at follow-up, using *t* tests for continuous measures and χ^2^ tests for categorical measures. Missing data in the risk factors were handled through multiple imputation. For each outcome, a single multivariate model that included all selected risk factors was run using generalized estimated equations to obtain risk ratios and corresponding 95% confidence intervals. For statistically significant risk factors, PAFs were calculated using Levin’s formula [16]. Because age and sex are nonmodifiable risk factors, PAFs were not calculated for them, irrespective of a statistically significant association with the outcomes.

## Results

**Fig. 1.**
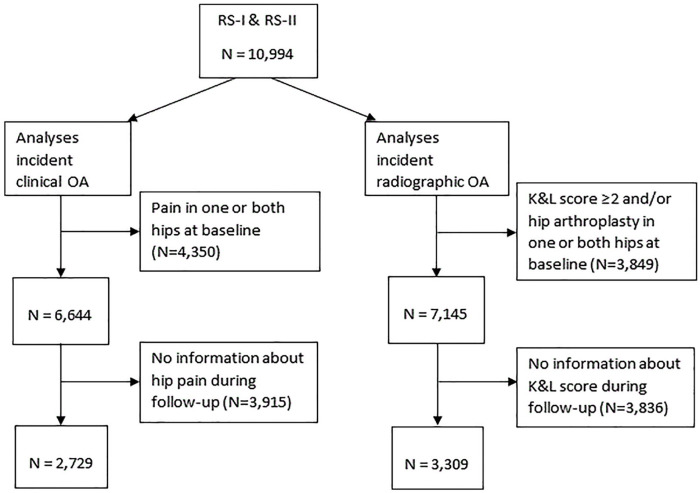
Flowchart showing the selection process for participants in RS-I and RS-II. Among the 10,994 participants of RS-I and RS-II, 2729 were chosen for analyses of incident clinical hip OA and 3309 were chosen for analyses on incident radiographic hip OA. *RS* Rotterdam study, *OA* osteoarthritis.

Of the 10,994 participants of RS-I and RS-II, 2729 were selected for the analyses on incident clinical hip OA and 3309 for the analyses on incident radiographic hip OA (Fig. 1, Table 1). Of the selected risk factors, age was significantly higher in those excluded for missing outcome data (68.1 ± 8.7 vs 64.6 ± 6.2; *P* < .001 in the analysis set for clinical hip OA and 69.5 ± 8.3 vs 63.1 ± 5.6; *P* < .001 in the analysis set for radiographic hip OA). Small but statistically significant differences in sex, BMI, smoking status, education level, diabetes, cam morphology, dysplasia, and CRP were deemed not clinically relevant.

**Table 1.** Baseline characteristics of selected participants in the Rotterdam study.

	Analysis set for clinical HOA	Analysis set for radiographic HOA
Sample size, N	2729	3309
Mean age (SD)	64.6 (6.2)	63.1 (5.6)
Sex, %		
Female	57.5	55.8
BMI category, %^a^		
Normal/underweight	38.3	36.3
Overweight	48.6	48.8
Obese	13.1	14.9
Smoking, %		
Current smoker	21.4	22.1
Former smoker	45.3	33.9
Never smoked	33.2	44.0
Education level, %		
Below average	58.8	63.7
Average	30.4	28.0
Above average	10.8	8.3
Diabetes, %	12.1	10.6
CRP, %		
≥10 mg/L	2.5	2.5
Cam morphology, % ^b^	11.4	10.8
Acetabular dysplasia, % ^b^	7.3	7.6
Radiographic thumb osteoarthritis, % ^c^	17.8	13.9
Radiographic hip osteoarthritis, % ^c^	4.7	0
Presence of hip pain, % ^b^	0	11.1

*BMI* body mass index, *CRP* c-reactive protein, *HOA* hip osteoarthritis, *K&L* Kellgren and Lawrence criteria.

a Normal/underweight: BMI ≤ 24.9. Overweight: BMI 25–29.9. Obese: BMI ≥ 30, ^b^ Present in 1 or both hips, ^c^ K&L grade ≥2 in 1 or both joints.

After a mean of 11.1 ± 0.6 years, the incidence of clinical hip OA was 19.9% (544/2729). After a mean of 10.9 ± 0.5 years, the incidence of radiographic hip OA was 9.9% (329/3309). Female sex, education level below average (relative to education level above average), and radiographic hip OA were statistically significantly associated with incident clinical hip OA (Table 2). Statistically significant risk factors for incident radiographic hip OA were female sex, age, overweight (relative to normal/underweight), and the presence of cam morphology, acetabular dysplasia, and radiographic thumb OA.

**Table 2.** Risk ratios from multivariate models for incident clinical and radiographic hip OA in the Rotterdam study.

Risk factors	Incident clinical hip OA^d^ risk ratio (95% CI)	Incident radiographic hip OA^e^ risk ratio (95% CI)
Sex, female	1.92(1.58–2.34)*	1.52 (1.190–1.94)*
Age	1.00 (0.99–1.01)	1.04 (1.02–1.06)*
Overweight^a^	1.00 (0.85–1.19)	1.51 (1.19–1.93)*
Obesity^a^	1.17 (0.93–1.46)	1.35 (0.97–1.88)
Current smoker^b^	0.96 (0.77–1.19)	1.23 (0.94–1.61)
Former smoker^b^	1.11 (0.93–1.33)	0.84 (0.65–1.08)
Below-average education level^c^	1.46 (1.05–2.04)*	1.00 (0.65–1.54)
Average education level^c^	1.33 (0.94–1.87)	1.03 (0.65–1.61)
Diabetes	1.10 (0.87–1.38)	1.17 (0.85–1.59)
CRP	1.43 (0.95–2.15)	1.52 (0.87–2.64)
cam morphology	1.17 (0.90–1.53)	1.80 (1.35–2.38)*
Acetabular dysplasia	1.28 (0.98–1.67)	1.49 (1.06–2.10)*
Radiographic thumb OA	1.05 (0.87–1.28)	1.35 (1.03–1.78)*
Radiographic hip OA	1.76 (1.35–2.28)*	—
Hip pain	—	1.21 (0.90–1.63)

*CI* confidence interval, *CRP* c-reactive protein, *OA* osteoarthritis.

Relative to: ^a^ “normal/underweight,” ^b^relative to “never smoker,” ^c^above average education level, ^d^analysis set for clinical HOA: N = 2729, ^e^analysis set for radiographic HOA: N = 3309. —: not included in the analyses.

Asterisks (*) mark statistically significant associations.

Based on the calculations of the PAFs, 21.4% of new cases of clinical hip OA were attributable to below-average education and 3.4% to radiographic hip OA. Overweight accounted for 20.0% of new cases of radiographic hip OA; cam morphology, for 7.9%; acetabular dysplasia, for 3.6%; and radiographic thumb OA, for 4.7%.

## Discussion

This study found an association between known risk factors, such as overweight, cam morphology, acetabular dysplasia, and radiographic thumb OA, and the development of radiographic hip OA in an open population of middle-aged and older adults. Uniquely, this study showed that only female sex, low education level, and baseline radiographic hip OA were associated with incident clinical hip OA after 11 years. Moreover, PAF analyses indicated that roughly 1 in 5 new cases of OA were attributable to education level (for clinical hip OA) and to overweight (for radiographic hip OA). Other significant risk factors accounted for less than 8% of new cases.

For incident clinical hip OA, a low education level was the most relevant risk factor, with ±20% of new cases attributable to it. Although rarely studied as a risk factor for clinical hip OA development, this association is not completely new. Higher level of education was previously reported to be associated with lower disease-specific pain scores and better physical function among subjects with radiographically diagnosed hip OA and hip symptoms [11] and with better patient-reported outcomes and quality of life after hip arthroplasty [5]. Low education level could well be a proxy for low health status [12], beyond factors like BMI, smoking, and diabetes, for which the analyses were adjusted. More research is required to confirm our results and to deepen our understanding of the causal factors behind the association between low education level and clinical hip OA incidence.

We found female sex to be the only risk factor statistically significantly associated with both incident clinical and radiographic hip OA. This might not be surprising, given the inconsistency in reported cross-sectional associations between hip symptoms and radiographic hip OA [3,8,10,19] and the low prevalence of radiographic hip OA in patients consulting for hip pain [21]. Recent systematic reviews showed that hip shape morphologies were strong risk factors for radiographic hip OA but also confirmed a lack of knowledge on the association between hip shape morphologies and clinical definitions of hip OA [4,25]. Our study found associations between hip shape morphology and radiographic hip OA development, but the contribution of these risk factors to new cases is much smaller (8% for cam morphology and 4% for acetabular dysplasia). Of note, the strength of these associations and their prevalence, together with the obtained PAFs, rely on the chosen cutoff values for the measurements [4]. Moreover, risk factors such as cam morphology and acetabular dysplasia are thought to lead to a rapid development of hip OA in younger people [1], a group that was potentially excluded in the current analyses due to the presence of hip pain and/or radiographic hip OA at baseline. For clinical hip OA development, our study found that cam morphology and acetabular dysplasia were not statistically associated with incident clinical hip OA in an unselected open–population cohort free of hip pain.

Our study has its limitations. First, despite the fact that certain local risk factors are associated with the risk of OA development in a joint, our analyses were performed on a person level. Although this might dilute potential associations, it is, from a primary preventive perspective, clinically relevant to assess potential treatment options for preventing hip OA at the person level rather than at the joint level. On the other hand, having unilateral hip OA increases the risk for bilateral hip OA; thus, these individuals would be an identifiable population for preventing OA in the unaffected joint. Second, not all known risk factors for hip OA development were incorporated in our analyses. For example, a family history of hip OA was not considered, given that this was not assessed in the RS-II data. Third, our definition of clinical hip OA was based purely on the presence of self-reported hip symptoms, which is similar but not identical to established clinical hip OA definitions, such as the National Institute for Health and Care Excellence (NICE) criteria [18].

In conclusion, our retrospective analysis of data from the Rotterdam Study found an association in a population of middle-aged and older individuals between low education level and incident clinical hip OA and between overweight and incident radiographic hip OA. Both risk factors accounted for 1 in 5 new cases of hip OA over 11 years of follow-up.

## Supplemental Material

sj-docx-1-hss-10.1177_15563316231192461 – Supplemental material for Risk Factors and Population-Attributable Fractions for Incident Hip Osteoarthritis Click here for additional data file. Supplemental material, sj-docx-1-hss-10.1177_15563316231192461 for Risk Factors and Population-Attributable Fractions for Incident Hip Osteoarthritis by Jos Runhaar, Annemaria C. van Berkel, Rintje Agricola, Joyce van Meurs and Sita M. A. Bierma-Zeinstra in HSS Journal®

sj-docx-2-hss-10.1177_15563316231192461 – Supplemental material for Risk Factors and Population-Attributable Fractions for Incident Hip Osteoarthritis Click here for additional data file. Supplemental material, sj-docx-2-hss-10.1177_15563316231192461 for Risk Factors and Population-Attributable Fractions for Incident Hip Osteoarthritis by Jos Runhaar, Annemaria C. van Berkel, Rintje Agricola, Joyce van Meurs and Sita M. A. Bierma-Zeinstra in HSS Journal®

sj-docx-3-hss-10.1177_15563316231192461 – Supplemental material for Risk Factors and Population-Attributable Fractions for Incident Hip Osteoarthritis Click here for additional data file. Supplemental material, sj-docx-3-hss-10.1177_15563316231192461 for Risk Factors and Population-Attributable Fractions for Incident Hip Osteoarthritis by Jos Runhaar, Annemaria C. van Berkel, Rintje Agricola, Joyce van Meurs and Sita M. A. Bierma-Zeinstra in HSS Journal®

sj-docx-4-hss-10.1177_15563316231192461 – Supplemental material for Risk Factors and Population-Attributable Fractions for Incident Hip Osteoarthritis Click here for additional data file. Supplemental material, sj-docx-4-hss-10.1177_15563316231192461 for Risk Factors and Population-Attributable Fractions for Incident Hip Osteoarthritis by Jos Runhaar, Annemaria C. van Berkel, Rintje Agricola, Joyce van Meurs and Sita M. A. Bierma-Zeinstra in HSS Journal®

sj-docx-5-hss-10.1177_15563316231192461 – Supplemental material for Risk Factors and Population-Attributable Fractions for Incident Hip Osteoarthritis Click here for additional data file. Supplemental material, sj-docx-5-hss-10.1177_15563316231192461 for Risk Factors and Population-Attributable Fractions for Incident Hip Osteoarthritis by Jos Runhaar, Annemaria C. van Berkel, Rintje Agricola, Joyce van Meurs and Sita M. A. Bierma-Zeinstra in HSS Journal®
